# Factors associated with food insecurity among the chronically ill population during the COVID-19 pandemic in the United States

**DOI:** 10.3389/fpubh.2023.1142603

**Published:** 2023-07-07

**Authors:** Caress A. Dean, Echu Liu, Kimberly R. Enard, Zhengmin Qian, Keith T. Elder

**Affiliations:** ^1^School of Health Sciences, Oakland University, Rochester, MI, United States; ^2^Department of Health Management and Policy, College for Public Health and Social Justice, Saint Louis University, Saint Louis, MO, United States; ^3^Department of Epidemiology and Biostatistics, College for Public Health and Social Justice, Saint Louis University, Saint Louis, MO, United States; ^4^Mississippi College, Clinton, MS, United States

**Keywords:** food insecurity, chronically ill, COVID-19, pandemic, United States

## Abstract

**Introduction:**

Little is known about food insecurity among Americans with chronic diseases, one of the vulnerable groups in health care. Factors influencing food insecurity among this population group are especially poorly understood.

**Methods:**

Using data from the COVID Impact Survey, this cross-sectional study sought to examine food insecurity among adults with chronic diseases in the United States and to identify factors associated with their risks for food insecurity during the COVID-19 pandemic.

**Results:**

Nearly 28% of the national and 32% of the regional samples from the COVID Impact Survey were at risk for food insecurity. The logistic regressions show that chronically ill US adults with one of the following characteristics have higher odds of being at risk for food insecurity: younger than 60 years, having financial stress, unemployed, having received food from a food pantry, without health insurance, having a household income lower than $100,000, and without a college degree.

**Discussion:**

Targeted policies and programs are warranted to address underlying determinants of food insecurity that adults with chronic illnesses experience.

## Introduction

A growing body of literature has identified a relationship between food insecurity and chronic conditions (1–5). For example, Gundersen and Ziliak’s study indicated that decreased nutrient intake is associated with fair or poor health and chronic illnesses such as diabetes, depression, and hypertension among non-senior adults (5). The literature has also shown lower food security related to the number of reported chronic conditions among working-age adults (2). Research has ascertained underlying factors of food insecurity among the chronically ill, in which the receipt of Supplemental Nutrition Assistance Program (SNAP) benefits has been identified as a determinant (2). Before the pandemic, studies showed that SNAP beneficiaries had a higher mortality rate for chronic conditions, such as cardiovascular disease and diabetes, compared to their non-SNAP counterparts (6–8). Their employment status may play a role in decreasing their food insecurity and accessing disease management services that may affect their mortality rate. However, research suggests that many SNAP recipients experience unstable employment (9). Specifically, a longitudinal study examining employment patterns among SNAP recipients determined that about two thirds of non-disabled SNAP recipients had unemployment periods within the study’s 3.5-year timeframe (9). Several studies have documented that SNAP beneficiaries who were not working reported their health, such as chronic conditions, as the reason (9, 10). These studies demonstrate the vulnerability of SNAP recipients with chronic diseases and food insecurity.

Unemployment and food insecurity have also been associated with being chronically ill among non-SNAP recipients (11). Previous research has identified the highest prevalence of chronic conditions among working-age adults who cannot work, followed by those who have been unemployed for at least 1 year, and then those unemployed for less than 12 months (12). Unemployment, along with the presence of chronic conditions, may create financial stress, which may affect adults’ health behaviors (13, 14). Multiple studies have reported that US adults with chronic illnesses have delayed care or do not adhere to their medication regimen due to cost concerns (15, 16). Their financial stress may also affect their dietary behavior, such as decreasing the consumption of healthy foods (13) and increasing the use of food pantries. A substantially high number of chronically ill US adults have been reported to utilize food pantries (17–20), with individuals having a high prevalence of modifiable conditions, including obesity, diabetes, hypertension, high cholesterol, heart disease, and stroke (20). With the increased use of food pantries among the chronically ill, disease management interventions have been implemented in food pantries to reach this population (19, 20). However, the COVID-19 pandemic may have affected this population’s use of these pantries.

Before the COVID-19 pandemic, food insecurity was one of the leading public health issues, with nearly 820 million people worldwide being food insecure in 2018 (21). The pandemic amplified this burden, as the United Nations reported in 2020 that 928 million people were severely food insecure (22), which may affect the diet and health outcomes of those with chronic conditions. Chronically ill individuals who experience food insecurity are a high-risk population, and the COVID-19 pandemic may have exacerbated their food insecurity. However, this relationship is understudied. Moreover, the pandemic has affected employment, SNAP benefits, and food pantry use, identified as factors associated with chronic condition status and food insecurity. Therefore, this study sought to address these gaps in the literature by examining food insecurity among adults with chronic diseases in the United States and identifying factors associated with their risks for food insecurity during the COVID-19 pandemic.

## Materials and methods

### Data source

This cross-sectional study used data from the COVID Impact Survey (23). The COVID Impact Survey was fielded by the National Opinion Research Center (NORC) at the University of Chicago over 3 weeks (April 20–26, 2020; May 4–10, 2020; and May 30–June 8, 2020). Data collected from these weeks are available for download on the survey’s website (23). We merged these three data sets for the analysis in this work.

One of the COVID Impact Survey’s critical aims was to generate national and regional statistics about various aspects of Americans’ lives during the pandemic; therefore, it surveyed a subset of the national and regional population to reflect accurately the larger group’s characteristics (4). The subset of the national population, the national sample hereafter, is from the NORC’s AmeriSpeak Panel (23), a panel of individuals selected from a 48-strata sampling based on age, race/Hispanic ethnicity, education, and gender (24). The subset of the regional population, the regional sample hereafter, includes adults from 18 regional areas, including 10 states (CA, CO, FL, LA, MN, MO, MT, NY, OR, and TX) and eight metropolitan statistical areas (Atlanta, Baltimore, Birmingham, Chicago, Cleveland, Columbus, Phoenix, and Pittsburgh) (23). These individuals were contacted via a U.S. Postal Service delivery-sequence file encompassing approximately 97% of US households (24).

The COVID Impact Survey was conducted in English and Spanish among US adults 18 years and older to examine their physical and mental health, economic status, and social systems (23). A total of 25,269 individuals completed the study. All participants in this survey received a monetary incentive (23). Additional details on the methodological approach of the COVID Impact Survey can be found on the Data Foundation’s website (25).

### Study sample

Our analysis focuses on those participants of all three waves of the COVID Impact Survey with at least one of the following chronic conditions: (1) diabetes; (2) high blood pressure or hypertension; (3) heart disease, heart attack, or stroke; (4) asthma; (5) chronic lung disease and COPD; (6) bronchitis and emphysema; (7) allergies; (8) a mental health condition; (9) cystic fibrosis; (10) liver disease or end-stage liver disease; (11) cancer; (12) a compromised immune system; or (13) overweight or obese. As a result, 4,964 of the national sample and 14,530 of the regional sample remained valid for analysis. After excluding those observations with missing responses to the survey questions used as the basis for constructing dependent and independent variables in this study, 4,809 of the national sample and 13,486 of the regional sample are used for the analysis.

### Measures

#### Dependent variable

The dependent variable of interest is a binary indicator constructed according to participants’ responses to the following two questions in the COVID Impact Survey: (1) “Please indicate whether the following statement was often true (=1), sometimes true (=2), or never true (=3) for you or your household in the past 30 days: We worried our food would run out before we got money to buy more.” (2) “Please indicate whether the following statement was often true (=1), sometimes true (=2), or never true (=3) for you or your household in the past 30 days: The food we bought did not last, and we did not have money to get more.” If a participant’s answer to the above two questions is “often true” or “sometimes true,” their households are at risk for food insecurity, and the dependent variable equals 1. Otherwise, the dependent variable equals 0 (26).

#### Independent variables

The following variables, created in line with the demographics or characteristics reported in the COVID Impact Survey, were considered as the potential predictors of being at risk for food insecurity: (1) age, (2) whether an individual respondent has financial stress, (3) whether an individual respondent has worked in the past 7 days, (4) whether an individual respondent has received SNAP or Food Stamps, (5) whether an individual respondent has received food from a food pantry, (6) whether an individual respondent has health insurance, (7) gender (whether an individual respondent is a female), (8) race (whether an individual respondent is a minority), (9) household income, (10) educational attainment, (11) household size, (12) census region, and (13) area of residence. Given that the responses to the COVID Impact Survey’s age, household income, and household size questions were recorded on an ordinal scale, age, household income, and household size are categorical variables.

Age is a variable with four categories: 18–29, 30–44, 45–59, and 60 or older. Financial stress is a binary indicator that equals 1 if a participant said they would need to cover an unexpected $400 expense by relying on one or more of the following outlets: (1) putting it on a credit card and paying it off over time; (2) money from a bank loan or line of credit; (3) borrowing from a friend or family member; (4) using a payday loan, deposit advance, or overdraft; (5) selling something, or (6) would not be able to pay for it right now.

Whether an individual respondent has received SNAP or Food Stamps is a binary indicator that equals 1 if they have received SNAP or Food Stamps when being interviewed. Whether an individual respondent has worked in the past 7 days, whether an individual respondent has received food from a food pantry, whether an individual respondent has health insurance, and gender are defined similarly. The COVID Impact Survey asks and solicits information about its respondents’ racial backgrounds by categorizing them into (1) White, non-Hispanic; (2) Black, non-Hispanic; (3) Hispanic; and (4) Other, non-Hispanic. Because non-White respondents account for a smaller percentage (less than 30%) of the national sample, we created a binary indicator of a minority that equals 1 if an individual respondent is non-white and 0 otherwise.

Based on the survey responses, we used five binary indicators to categorize our sample’s household income: less than $10,000; $10,000 to $29,999; $30,000 to $49,999; $50,000 to $99,999; and higher than $100,000. Because educational attainment critically determines income, we also considered the respondent’s highest level of education by including the following four dichotomous variables in our analysis: less than high school, high school, some college, and a bachelor’s degree or higher. Household size is also associated with household income; therefore, we used six dummy variables to categorize our study sample’s household size according to data the COVID Impact Survey collected: one, two, three, four, five, and six or more.

Income levels reportedly varied across regions (27), so the four dummies, equal to 1 if a survey respondent’s household is in the Northeast, Midwest, South, or West region, were included in our analysis as potentially independent variables. People living in some urban and rural areas may have limited access to full-service supermarkets or grocery stores (28); therefore, a variable defining area of residence (urban, suburban, and rural) was also included.

#### Statistical analysis

Our statistical analysis consists of three steps. First, we summarize our data by creating a frequency table (Table 1). Second, we summarize the characteristics of our study sample by their food insecurity status and test whether the features are independent of food insecurity status (Tables 2, 3). Third, multivariate logistic regressions were used to estimate the odds of being at risk for food insecurity. Any variable having a significant univariate test at a 5% level in the previous step was selected as potential independent variables for the multivariate analysis. The variance inflation factor (VIF) was computed for these predictors chosen before they were included in the regressions to ensure the non-existence of multicollinearity. Sampling weights from the COVID Impact Survey were considered when performing the regressions, and a *p* < 0.05 was considered the significant level for statistical tests. All analyses were conducted using STATA 15.1.

**Table 1:** Frequency of characteristics: national and regional samples.

Variables	National sample*		Regional sample^†^	
	*n*	%^‡^	*n*	%^‡^
**Being at risk for food insecurity**				
Yes	1,305	28.72	2,484	31.68
No	3,504	71.28	11,002	68.32
**Age**				
18–29	566	17.51	1,464	18.35
30–44	1,346	23.86	2,854	25.68
45–59	1,153	25.05	3,238	23.46
60+	1,744	33.58	5,930	32.51
**Having financial stress**				
Yes	2,097	45.87	4.947	51.87
No	2,712	54.13	8,539	48.13
**Working in the past 7 days**				
Yes	2,296	46.84	6,692	46.12
No	2,513	53.16	6,794	53.88
**Having received SNAP or food stamps**				
Yes	581	11.29	1,043	13.90
No	4,228	88.71	12,443	86.10
**Having received food from a food pantry**				
Yes	358	7.60	644	8.50
No	4,451	92.40	12,842	91.50
**Covered by health insurance**				
Yes	4,480	92.82	12,834	89.60
No	329	7.18	652	10.40
**Being a female**				
Yes	2,538	53.36	7,889	52.75
No	2,271	46.64	5,597	47.25
**Being a minority**				
Yes	1,633	34.81	2,947	40.26
No	3,176	65.19	10,539	59.74
**Household income**				
Less than $10,000	256	5.65	664	9.03
Between $10,000 and $29,999	1,013	21.14	2,114	22.63
Between $30,000 and $49,999	962	19.52	2,199	17.54
Between $50,000 and $99,999	1,587	31.56	4,427	28.18
More than $100,000	991	22.12	4,082	22.63
**Education**				
Less than high school	240	9.73	357	8.56
High school	865	27.15	1,407	27.66
Some college	2,045	28.98	3,832	31.59
College or above	1,659	34.13	7,890	32.19
**Household size**				
One	1,516	35.20	4,183	28.01
Two	1,346	25.67	5,200	33.03
Three	688	13.72	1,862	16.04
Four	479	9.64	1,340	11.63
Five	308	5.96	584	6.67
Six or more	472	9.82	317	4.63
**Census region**				
Northeast	709	18.01	1,534	14.76
Midwest	1,212	21.03	3,889	18.67
South	1,695	38.47	4,210	36.94
West	1,193	22.49	3,853	29.62
**Are of residence**				
Urban	3,474	70.39	10,816	81.78
Suburban	935	20.19	2,001	13.72
Rural	400	9.42	669	4.51

* *N* = 4,809.

† *N* = 13,486.

‡ Weighted percentages.

**Table 2:** Characteristics of the national sample* by the risk status of food insecurity.

Variables	Being at risk for food insecurity	Not being at risk for food insecurity	*p* value
Age (%^‡^)			0.00
18–29	212 (25.87)	354 (14.14)	
30–44	475 (32.52)	871 (20.37)	
45–59	325 (22.64)	828 (26.02)	
60+	293 (18.97)	1,451 (39.47)	
Having financial stress (%^‡^)			0.00
Yes	1,050 (82.03)	1,047 (31.29)	
No	255 (17.97)	2,457 (68.71)	
Working in the past 7 days (%^‡^)			0.00
Yes	502 (38.92)	1,794 (50.04)	
No	803 (61.08)	1,710 (49.96)	
Having received SNAP or Food Stamps (%^‡^)			0.00
Yes	353 (25.80)	228 (5.45)	
No	952 (74.20)	3,276 (94.55)	
Having received food from a food pantry (%^‡^)			0.00
Yes	245 (18.99)	113 (3.01)	
No	1,060 (81.01)	3,391 (96.99)	
Covered by health insurance (%^‡^)			0.00
Yes	1,139 (87.39)	3,341 (95.01)	
No	166 (12.61)	163 (4.99)	
Being a female (%^‡^)			0.00
Yes	774 (59.99)	1,764 (50.68)	
No	531 (40.01)	1,740 (49.32)	
Being a minority (%^‡^)			0.00
Yes	665 (46.30)	968 (30.17)	
No	640 (53.70)	2,536 (69.83)	
Household income (%^‡^)			0.00
Less than $10,000	156 (13.46)	100 (2.51)	
Between $10,000 and $29,999	459 (34.08)	554 (15.93)	
Between $30,000 and $49,999	301 (20.89)	661 (18.97)	
Between $50,000 and $99,999	301 (24.17)	1,286 (34.54)	
More than $100,000	88 (7.40)	903 (28.05)	
Education (%^‡^)			0.00
Less than high school	128 (18.87)	112 (6.05)	
High school	317 (34.76)	548 (24.09)	
Some college	606 (30.10)	1,439 (28.53)	
College or above	254 (16.27)	1,405 (41.33)	
Household size (%^‡^)			0.00
One	356 (30.92)	1,160 (36.93)	
Two	258 (17.98)	1,088 (28.76)	
Three	199 (15.00)	489 (13.20)	
Four	155 (11.53)	324 (8.88)	
Five	126 (7.59)	182 (5.30)	
Six or more	211 (16.98)	261 (6.93)	
Census region (%^‡^)			0.48
Northeast	183 (19.25)	526 (17.51)	
Midwest	300 (19.59)	912 (21.61)	
South	516 (39.67)	1,179 (37.98)	
West	306 (21.48)	887 (22.90)	
Are of residence (%^‡^)			0.47
Urban	926 (69.00)	2,548 (70.95)	
Suburban	249 (20.48)	686 (20.08)	
Rural	130 (10.53)	270 (8.98)	

* *N* = 4,809.

‡ Weighted percentages.

**Table 3:** Characteristics of the regional sample* by the risk status of food insecurity.

Variables	Being at risk for food insecurity	Not being at risk for food insecurity	*p* value
Age (%^‡^)			0.00
18–29	492 (28.26)	972 (13.76)	
30–44	735 (31.51)	2,119 (22.98)	
45–59	676 (22.10)	2,562 (24.08)	
60+	581 (18.12)	5,349 (39.18)	
Having financial stress (%^‡^)			0.00
Yes	2,115 (88.78)	2,832 (34.75)	
No	369 (11.22)	8,170 (65.25)	
Working in the past 7 days (%^‡^)			0.00
Yes	943 (33.48)	5,749 (51.99)	
No	1,541 (66.52)	5,253 (48.01)	
Having received SNAP or Food Stamps (%^‡^)			0.00
Yes	676 (31.12)	367 (5.92)	
No	1,808 (68.88)	10,635 (94.08)	
Having received food from a food pantry (%^‡^)			0.00
Yes	441 (20.76)	203 (2.81)	
No	2,043 (79.24)	10,799 (97.19)	
Covered by health insurance (%^‡^)			0.00
Yes	2,145 (79.53)	10,689 (94.28)	
No	339 (20.47)	313 (5.72)	
Being a female (%^‡^)			0.00
Yes	1,784 (64.23)	6,105 (47.43)	
No	700 (35.77)	4,897 (52.57)	
Being a minority (%^‡^)			0.00
Yes	1,070 (57.83)	1,877 (32.11)	
No	1,414 (42.17)	9,125 (67.89)	
Household income (%^‡^)			0.00
Less than $10,000	434 (21.57)	230 (3.21)	
Between $10,000 and $29,999	903 (40.60)	1,211 (14.30)	
Between $30,000 and $49,999	539 (19.00)	1,660 (16.86)	
Between $50,000 and $99,999	483 (15.52)	3,944 (34.04)	
More than $100,000	125 (3.31)	3,957 (31.58)	
Education (%^‡^)			0.00
Less than high school	233 (19.15)	124 (3.65)	
High school	548 (38.36)	859 (22.70)	
Some college	1,016 (32.27)	2,816 (31.28)	
College or above	687 (10.22)	7,203 (42.38)	
Household size (%^‡^)			0.00
One	761 (23.80)	3,422 (29.96)	
Two	650 (22.76)	4,550 (37.79)	
Three	425 (19.96)	1,437 (14.22)	
Four	293 (12.26)	1,047 (11.34)	
Five	205 (11.83)	379 (4.28)	
Six or more	150 (9.40)	167 (2.41)	
Census region (%^‡^)			0.01
Northeast	291 (16.14)	1,243 (14.12)	
Midwest	626 (15.97)	3,263 (19.93)	
South	943 (39.56)	3,267 (35.73)	
West	624 (28.33)	3,229 (30.22)	
Are of residence (%^‡^)			0.07
Urban	1,931 (79.52)	8,885 (82.84)	
Suburban	393 (15.47)	1,608 (12.89)	
Rural	160 (5.01)	509 (4.27)	

* *N* = 13,486.

‡ Weighted percentages.

## Results

Approximately 29% of the national sample in our analysis reported being at risk for food insecurity (Table 1). This percentage was slightly higher among the regional sample, with over 31% reporting being at risk for food insecurity. The age distributions of the national and regional samples included in this study are similar, but the national sample has a slightly higher percentage of individuals over 45 years old. A more significant portion of the regional sample (51.87%) reported experiencing financial stress than the national sample (45.87%) did. A higher percentage of the regional sample, compared to the national sample, reported receiving SNAP or Food Stamps (13.90% vs. 11.29%). The same conclusion is applied to food pantry assistance (8.50% vs. 7.60%).

Table 1 also reports that most national and regional samples included in this study have health insurance coverage (92.82 and 89.60%, respectively). Additionally, the regional sample has more minorities than the national sample (40.26% vs. 34.81%) does. Both national and regional samples, as Table 1 indicates, have a similar household income distribution; most households have income between $50,000 and $99,999. Table 1 also shows that most national and regional samples included in this study have a high school or above degrees and live in the south and urban areas. The household size distributions for national and regional samples differ, as Table 1 indicates. For the national sample, households with a single person have the highest frequency, while two-people households have the highest frequency for the regional sample.

As Table 2 demonstrates, whether an individual in the national sample included in this study is at risk for food insecurity differs according to their ages, financial stress status, working status, the status of receiving public assistance (SNAP or Food Stamps), the status of receiving food assistance (food pantry), health insurance status, gender, race, household income, educational attainment, and household size. Table 3 shows whether an individual in the regional sample included in our analysis is at risk for food insecurity, which differs by the same set of variables and the US census region. The VIF value for these variables ranges between 1.04 and 3.10 for the national sample and 1.05 and 3.53 for the regional sample. Therefore, multicollinearity does not seem to be a concern for including these variables as predictors in the logistic regressions.

Table 4 depicts the association between those predictor variables identified from Table 2 and the odds of being the risk for food insecurity among the national sample included in our study. According to this table, the odds for the national sample aged at least 60 years old to be at risk of having food shortage is 0.41 times that of those who are under 60 (odds ratio [OR] = 0.41, 95% Confidence Interval [CI]: 0.28–0.61). In addition, financially stressed respondents’ odds of experiencing food shortage are 5.54 times that of those who are not economically stressed (OR = 5.54, 95% CI: 4.37–7.03). Compared to those who did not work in the past 7 days, respondents who worked in the past 7 days had lower odds of being at risk of running out of food (OR = 0.72, 95% CI: 0.56–0.93). Regarding the use of food assistance services, the national sample in our study who reported receiving SNAP or Food Stamps (OR = 1.49, 95% CI:1.08–2.06) or receiving food at a food pantry (OR = 3.26, 95% CI:2.13–5.00) had significantly higher odds of being at risk for food insecurity.

**Table 4:** Estimated odds ratios from the logistic regression using the national sample.*

Characteristics	Estimate	SE^†^	95% CI^‡^		*p*
			LL^§^	UL^§^	
**Age**					
18–29 (reference)					
30–44	1.24	0.22	0.88	1.75	0.23
45–59	0.75	0.14	0.52	1.08	0.12
60+	0.41	0.08	0.28	0.61	0.00
**Having a financial stress**					
Yes	5.54	0.67	4.37	7.03	0.00
No (reference)					
**Working in the past 7 days**					
Yes	0.72	0.09	0.56	0.93	0.01
No (reference)					
**Having received SNAP or food stamps**					
Yes	1.49	0.25	1.08	2.06	0.02
No (reference)					
**Having received food from a food pantry**					
Yes	3.26	0.71	2.13	5.00	0.00
No (reference)					
**Covered by health insurance**					
Yes	0.66	0.13	0.46	0.96	0.03
No (reference)					
Being a female	1.11	0.13	0.88	1.39	0.39
Being a minority	1.20	0.14	0.95	1.52	0.13
**Household income**					
Less than $10,000	6.17	1.99	3.28	11.62	0.00
Between $10,000 and $29,999	3.24	0.69	2.14	4.90	0.00
Between $30,000 and $49,999	2.31	0.47	1.55	3.45	0.00
Between $50,000 and $99,999	1.91	0.36	1.32	2.76	0.00
More than $100,000 (reference)					
**Education**					
Less than high school	2.23	0.56	1.36	3.66	0.00
High school	1.33	0.22	0.97	1.83	0.08
Some college	1.39	0.19	1.07	1.81	0.01
College or above (reference)					
**Household size**					
One (reference)					
Two	1.07	0.17	0.79	1.46	0.67
Three	1.49	0.28	1.03	2.14	0.03
Four	1.43	0.28	0.97	2.09	0.07
Five	1.41	0.33	0.88	2.24	0.15
Six or more	2.37	0.51	1.56	3.60	0.00

* *N* = 4,809.

† SE, standard error.

‡ CI, confidence interval.

§ LL, lower limit; UL, upper limit.

Table 4 also shows that for national samples with health insurance coverage in our study, their odds of being at risk for food insecurity is 0.66 times that of those without (OR = 0.66, 95% CI: 0.46–0.96). Compared to survey respondents with a household income of more than $100,000, the odds of being at risk for food insecurity are significantly higher for those with less household income, as Table 4 indicates. In general, respondents with less educational attainment and a larger household size were estimated to have higher odds of being at risk of running out of food, based on the estimated ORs reported in Table 4.

Similar to the results from the analysis of the national sample reported in Tables 4, 5 shows being at risk of food insecurity is more common among those who are financially stressed (OR = 5.25, 95% CI: 4.18–6.59) and have food from a food pantry (OR = 3.47, 95% CI: 2.38–5.64). Additionally, those aged at least 60 years old in the regional sample for our analysis have lower odds of being at risk for a food shortage (OR = 0.46, 95% CI: 0.33–0.65). Furthermore, for non-White respondents in our regional sample data, the odds of being at risk of food shortage is 1.45 times that of White respondents, according to Table 4 (OR = 1.45, 95% CI: 1.16–1.80). In contrast, the regional sample aged over 60 years, working in the past 7 days, having health insurance, having a household income of more than $100,000, having a college education, or households with only one member in the household included in this study has lower odds of being at risk for food insecurity.

**Table 5:** Estimated odds ratios from the logistic regression using the regional sample.*

Characteristics	Estimate	SE^†^	95% CI^‡^		*p*
			LL^§^	UL^§^	
**Age**					
18–29 (reference)					
30–44	1.29	0.21	0.93	1.77	0.12
45–59	0.90	0.16	0.64	1.26	0.53
60+	0.46	0.08	0.33	0.65	0.00
**Having a financial stress**					
Yes	5.25	0.61	4.18	6.59	0.00
No (reference)					
**Working in the past 7 days**					
Yes	0.77	0.09	0.61	0.98	0.03
No (reference)					
**Having received, applied for, or tried to apply for SNAP**					
Yes	1.25	0.21	0.90	1.75	0.19
No (reference)					
**Having received food from a food pantry**					
Yes	3.47	0.66	2.38	5.04	0.00
No (reference)					
**Covered by health insurance**					
Yes	0.57	0.11	0.39	0.83	0.00
No (reference)					
Being a female	1.17	0.13	0.94	1.45	0.16
Being a minority	1.45	0.16	1.16	1.80	0.00
**Household income**					
Less than $10,000	13.84	3.85	8.02	23.89	0.00
Between $10,000 and $29,999	9.54	2.15	6.13	14.84	0.00
Between $30,000 and $49,999	5.66	1.22	3.71	8.63	0.00
Between $50,000 and $99,999	2.84	0.59	1.89	4.27	0.00
More than $100,000 (reference)					
**Education**					
Less than high school	3.23	0.76	2.03	5.13	0.00
High school	1.89	0.28	1.41	2.53	0.00
Some college	1.63	0.20	1.28	2.08	0.00
College or above (reference)					
**Household size**					
One (reference)					
Two	1.43	0.20	1.09	1.87	0.01
Three	1.99	0.36	1.39	2.85	0.00
Four	1.49	0.29	1.01	2.20	0.04
Five	3.73	0.86	2.37	5.85	0.00
Six or more	2.54	0.74	1.44	4.48	0.00
**Census region**					
Northeast	1.16	0.21	0.82	1.64	0.41
Midwest	0.95	0.12	0.74	1.21	0.68
South (reference)					
West	0.98	0.13	0.75	1.28	0.88

* *N*, 13,486.

† SE, standard error.

‡ CI, confidence interval.

§ LL, lower limit; UL, upper limit.

In summary, our logistics regression results show that, among the chronically ill US population, those who are older, work, have health insurance coverage, have higher income, or are more educated tend to have lower odds of a food shortage. Conversely, people with financial challenges or who rely on food assistance programs tend to have higher odds of a food shortage.

## Discussion

The COVID-19 pandemic increased food insecurity, which may affect the diet and health outcomes of the chronically ill. This cross-sectional study determined that, among the national sample of the COVID Impact Survey, those who were chronically sick and received SNAP benefits or food from a food pantry had a higher risk of running out of food. However, no studies have examined the relationship between SNAP benefits, chronic illnesses, and food insecurity during the pandemic nationally. One prior study found that receiving SNAP benefits significantly associated with food insecurity among adults aged 65 and older during the pandemic (11). The same study also found a high prevalence of chronic conditions among older adults who were food insecure (11). These findings highlight the importance of ensuring access to food assistance programs among the chronically ill during public health emergencies.

Note that the relationship between SNAP benefits and food security risk among the regional sample of chronically ill participants was not statistically significant. State-level differences in SNAP benefits may have influenced this relationship. Additional research should be conducted to understand the influence of geographic location on receiving SNAP benefits for the chronically ill during the pandemic.

Among the national and regional samples, the odds of reporting financial stress were five times higher among chronically ill participants at risk of food insecurity than their non-risk counterparts. These findings are disconcerting as previous studies have linked delayed care and nonadherence to treatment regimens to cost concerns (15, 16) and economic pressure to consume an unhealthy diet (13, 14). As a result, chronically ill individuals’ conditions may have worsened. Further research should be performed to assess the impact of this population’s financial stress during the pandemic on health behaviors and determine the implications of their financial stress on their current and future disease status.

In the national and regional samples, chronically ill participants who did work in the past 7 days had significantly lower odds of being at risk for food insecurity. Similarly, sick, chronically participants who reported having health insurance coverage had significantly lower odds of being at risk for food insecurity. No studies have examined job loss among the chronically ill during the pandemic. However, an unprecedented number of job and wage losses did occur due to the stay-at-home orders (14, 15) and may have affected those with chronic conditions. Additionally, previous research has found chronically ill US adults are more likely to have some form of health insurance (29). Therefore, the association with no coverage among chronically ill participants with food insecurity risk may stem from the high unemployment rate and loss of employer-sponsored health coverage. This theory is supported by a study that determined 2.7 million Americans lost health insurance during 12 weeks of the pandemic (30). However, this study did not focus on the chronically ill population. Therefore, future research should be conducted to ascertain health insurance loss among the sick chronically at risk of food insecurity during the pandemic.

This study has limitations to consider. The study utilized a cross-sectional design; therefore, a causal relationship cannot be determined. Participants self-reported their responses, allowing for recall bias. This study is also subjected to selection bias due to online and telephone interviews. Data collection occurred from March 2020 to June 2020. It utilized different sample populations during each data collection point, which prohibits understanding food security of the chronically ill over time during the pandemic. The data do not contain information on the food insecurity risk among participants before the pandemic; therefore, this study cannot determine the direct effect of COVID-19 on food insecurity among the chronically ill.

A growing body of literature describes the complex and bidirectional relationship between food insecurity and chronic illnesses (12, 13, 19). However, little is known about the effect of COVID-19 on this relationship. This study’s outcomes address this void because it identified factors associated with a risk of food insecurity among the chronically ill during the pandemic. These findings can be utilized in future research to inform targeted policies and programs to support this vulnerable population and to ensure access to food assistance programs and health insurance during future public health emergencies.

## Data availability statement

The original contributions presented in the study are included in the article/supplementary materials, further inquiries can be directed to the corresponding author.

## Author contributions

KRE, ZQ, and KTE contributed to the study’s conception and design. EL and CAD organized the database and wrote the first and second drafts of the manuscript. EL performed the statistical analysis. CAD, KRE, ZQ, and KTE reviewed and revised all manuscript sections. All authors contributed to the article and approved the submitted version.

## Conflict of interest

The authors declare that the research was conducted in the absence of any commercial or financial relationships that could be construed as a potential conflict of interest.

## Publisher’s note

All claims expressed in this article are solely those of the authors and do not necessarily represent those of their affiliated organizations, or those of the publisher, the editors and the reviewers. Any product that may be evaluated in this article, or claim that may be made by its manufacturer, is not guaranteed or endorsed by the publisher.
